# Migraine symptoms and association of triggers, coping strategies and clinical characteristics with COVID-19 diagnosis among university students in Peninsular Malaysia: A cross-sectional study

**DOI:** 10.51866/oa.521

**Published:** 2024-07-31

**Authors:** Selvakumar Kiruthika, Lee Fan Tan, Chai Nien Foo

**Affiliations:** 1 BPT, PT (Neuro Sciences), Department of Physiotherapy, M. Kandiah Faculty of Medicine and Health Sciences, Universiti Tunku Abdul Rahman, Selangor, Malaysia. Email: kiruthika@utar.edu.my; 2 BE (Biomedical Engineering), PhD (Engineering), Department of Mechatronics and BioMedical Engineering, Lee Kong Chian Faculty of Engineering and Science, Universiti Tunku Abdul Rahman, Selangor, Malaysia.; 3 BSc (Food Science), MBA (General Management), PhD (Community Health), Department of Population Medicine, M. Kandiah Faculty of Medicine and Health Sciences, Universiti Tunku Abdul Rahman, Selangor, Malaysia.

**Keywords:** Migraine disorders, COVID-19, Pandemic, triggers, Coping strategies

## Abstract

**Introduction:**

Differentiating between migraine and COVID-19 headaches is essential for better treatment. Evidence-based research during the COVID-19 pandemic has found that university students are more likely to experience migraine. Migraine can affect academic performance, sleep pattern, social and emotional well-being if left untreated or misdiagnosed. This study aimed to determine the prevalence of migraine symptoms and the association of triggers, coping strategies and clinical characteristics with COVID-19 diagnosis.

**Methods:**

This cross-sectional study was conducted across higher educational institutions in Peninsular Malaysia. Convenience sampling was applied to recruit full-time university students. A reliable and validated instrument was used to evaluate demographic data, migraine symptoms, triggers, coping strategies and clinical characteristics of migraine (frequency, intensity, severity and duration) during COVID-19 diagnosis.

**Results:**

The response rate was 98.3%, where 485 out of 493 responses were analysed. The prevalence of migraine was 35.9% (n=174). None of the triggers, coping strategies and clinical characteristics of migraine were significantly associated with COVID-19 diagnosis.

**Conclusion:**

The university students in Peninsular Malaysia showed a considerable prevalence of migraine symptoms. During the pandemic, the common triggers for existing symptoms were stress and a lack of sleep (combined triggers). The coping strategy adopted by most of the university students was lifestyle changes and in the COVID-19 positive group maladaptive coping strategies were adopted indicating the need for further investigation.

## Introduction

The COVID-19 pandemic has significantly transformed the lifestyles of people worldwide, indicating the need for an immediate adaptation to the new situation.^1^ The World Health Organization examined more than 55,000 confirmed cases of COVID-19 and found that headache was present in 13.6% of these cases. Headache can also be a symptom of COVID-19 in people with migraine. Therefore, differentiating between migraine and COVID-19 headaches is essential for better treatment. A migraine attack is a primary headache due to changes in nerve signalling or levels of neurotransmitters such as serotonin, whereas a COVID-19 headache is a secondary headache caused by another underlying disease or condition.^2^ Research has indicated that headache caused by the coronavirus occurs simultaneously with newly experienced fatigue and loss of smell. It may appear as moderate-to-severe, pressing, pulsating or stabbing pain, usually felt equally on both sides of the head instead of only one central area and lasting for >3 days.^3^ Studies have analysed the impact of the COVID-19 pandemic on migraine symptoms. However, these studies have been conducted among general populations rather than on a specific age group. Recent research has reported that students’ physical, mental and social well-being and academic performance are affected.^4,5^ This highlights that university students are especially vulnerable during this worldwide health catastrophe.^6^ Some studies have indicated that migraine is more prevalent among university students.^7,8^ If left untreated or undiagnosed, migraine can impact academic performance, sleep pattern, attention span during lectures and social and emotional well-being. In this population, the introduction of particular stressors and lifestyle modifications caused by the transition to higher education may affect the frequency and severity of migraine symptoms.^9^ The characteristics of migraine symptoms also vary during the pandemic. Latin American studies have shown that 48.6% of patients with migraine experience worsened symptoms; 15.6%, improved symptoms; and 35.8%, unchanged symptoms.^10^ Patients with a history of migraine who have recovered from COVID-19 have shown an increased frequency of migraine attacks and anxiety.^11^ The COVID-19 pandemic has had an overall negative impact on patients with migraine. After the emergence of COVID-19, the majority of patients with COVID-19 with previous headaches have reported that their new emerging headaches during the infection period differ from their usual headaches,^12^ indicating the need to analyse the prevalence and triggers of and coping strategies for migraine. Beyond the academic and social spheres, the pandemic also affects students’ overall well-being, which includes physical health. The persistent danger of COVID-19 contagion, less human interactions, virtual learning and financial instability are only some of the difficulties faced by university students.^13^ These adversities may impact not only their susceptibility to headaches but also the progression and manifestation of their existing migraine symptoms.

One study in Malaysia analysed the impact of the COVID-19 pandemic on coping strategies and the effect of the movement control order. The results revealed that about 30% of students experienced some level of anxiety due to the COVID-19 pandemic and that students used maladaptive coping strategies more than adaptive coping strategies to deal with anxiety caused by the pandemic and the effect of movement restriction.^14^ Simultaneously, a study also suggested monitoring students’ mental health status during the pandemic.^15^

Despite efforts to analyse coping strategies adopted during the pandemic, there remains a lack of age-specific data and outcome measures for assessing the impact of the pandemic on existing migraine symptoms. Addressing these gaps is vital for tailored interventions to mitigate the pandemic’s impact on university students’ well-being and academic success. This study aimed to determine the prevalence of migraine symptoms and the association of triggers, coping strategies and clinical characteristics with COVID-19 diagnosis using a validated and reliable instrument. The phases of COVID-19 diagnosis included confirmed diagnosis, suspected exposure and absence of diagnosis. The main objectives of this study were to assess the prevalence of migraine among university students and identify the association of triggers, coping strategies and clinical characteristics with COVID-19 diagnosis. The findings of this study may help academic and educational trainers understand the current status of students and plan targeted interventions.

## Methods

### Procedures

**Figure 1:**
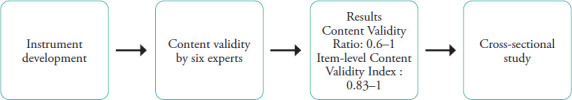
Flow of the study.

This is a cross-sectional online study. Students from both public and private universities in Peninsular Malaysia participated in this study. A questionnaire was developed following content validity analysis and administered online on social media platforms such as WhatsApp and Facebook and via e-mail. Data were collected from December 2021 to April 2022. Participants were briefed on the purpose of the study, and consent was obtained from those willing to participate. Participants were assured that their participation in this study was completely voluntary and that their responses would be anonymous. The sample size was calculated based on facts and figures from the Malaysia Ministry of Education (MoE) and Ministry of Higher Education (MoHE) rightsizing in 2020.^16^ The estimated sample size was determined using Krejcie and Morgan’s table to account for a 5% margin of error, a 95% confidence interval and a 50% response distribution. The sample size was increased to 482 to accommodate for a potential 20% non-response rate.^17^ Full-time university students of both sexes aged from 18 to 40 years were included. Conversely, university students pursuing part-time degrees and unwilling to participate in this study were excluded. Figure 1 summarises the flow of this study.

### Study instrument

A detailed questionnaire for assessing the prevalence and triggers of and coping strategies for migraine was developed based on the literature review. The new scale developed was based on a three-stage analysis: instrument development, judgement based on expert opinion from various fields and content validity analysis.^18^ The final questionnaire consisted of 11 questions. A web-based technical design was planned, and this online questionnaire consisted of eight sections. Section 1 briefed participants on the purpose of the study, informed consent and the Personal Data Protection Act; Section 2 collected demographic details; and Sections 3–8 required participants to respond based on their COVID-19 [polymerase chain reaction (PCR)] results.

The questionnaire was content validated through an online review by six experts. These experts were neuro-physiotherapists, language and linguistics experts and psychologists. Based on the assessment, each item’s relevance was evaluated: The item-level content validity index (I-CVI) ranged from 0.83 to 1. The average I-CVI of the overall scale was 0.92. The scale-level content validity indexuniversal agreement (S-CVI/UA) was 0.54, and the scale-level content validity indexaverage (S-CVI/Ave) based on the I-CVI was 0.92. The scale-level content validity index (S-CVI) based on the proportion relevance was 0.92, similar to the I-CVI. The number of experts (n=6) was considered adequate for content validation, as the number was within 3–10.^19^ The normal value indicating excellent content validity for the I-CVI is 0–1 ; S-CVI/UA, ≥0.8; and S-CVI/Ave, ≥0.9.^20^ The S-CVI/UA method may underestimate the content validity of the overall questionnaire since the likelihood of achieving 100% agreement in all items decreases when the number of experts increases.^21^ The content validity index relative to clarity for each item ranged from 0.83 to 1. These results indicated that the scale had a high content validity. The content validity ratio (CVR) for each item ranged from 0.6 to 1, indicating that half or a more significant number of panellists rated the items to be essential for the construct of migraine symptoms during the COVID-19 pandemic.

### Demographic and academic data

The demographic and academic data collected included age, sex, area of study, name of institution, current level of study and year of study. Participants were asked whether they experienced migraine. A brief definition of migraine was also provided.^22^ Additionally, the time of onset of symptoms, frequency of pain per week and month and intensity of pain were assessed. Based on the subjective PCR results (diagnosed/non-diagnosed and close contact/suspected to be in close contact during periods of infection), participants were requested to answer the respective section.

### Triggers of migraine

Migraine triggers are common and divided into five main categories: emotional stress, menstruation, sleep disturbance, food, alcoholic beverages and weather changes. Based on previous studies about migraine triggers, the triggers included were social isolation, side effects of drugs consumed, wearing of a mask for longer periods, the infection itself, stress, a lack of sleep, fatigue, inadequate food intake, physical exercise, hormonal changes, cold weather, exposure to sunlight for a long period, alcohol, various sensory stimuli and other factors.^12^

### Coping strategies for migraine

Coping strategies are generally classified into adaptive and maladaptive. Adaptive coping strategies include active coping, problem-solving and social support seeking. Maladaptive coping strategies include avoidance and self-blaming, which are more prevalent among young adults. Based on previous studies, the coping strategies included hospitalisation, COVID-19 medications, migraine medications, adequate sleep and exercise, diet modification, relaxation exercise, social support seeking, acceptance, mental disengagement and humanitarianism.^23^

### Clinical characteristics of migraine

Based on the literature, the clinical factors included were the intensity, severity, frequency and duration of pain since the start of the pandemic. Hence, upon fulfilment of preliminary validity, the questionnaire was used to conduct the cross-sectional study.

### Data analysis

Data were analysed using IBM SPSS Statistics 22.0 (USA). Quantitative variables were summarised as means and standard deviations. Qualitative variables were described as frequencies and percentages. The chi-square test was used for further analysis. The level of significance was set at P<0.05 for all tests.

## Results

A total of 493 participants were enrolled in the study. Among them, 485 were included for further analysis. Table 1 summarises the demographic and academic data of the participants.

**Table 1:** Demographic data of the participants (N=485).

Variables	Migraine, n (%)	
	Yes (n=174) n (%)	No (n=311) n (%)
**Sex**		
Female	124 (71.3)	205 (65.9)
Male	47 (27.0)	100 (32.1)
Prefer not to say	3(1.7)	6(1.9)
**Age, year**		
18-20	63 (36.2)	132 (42.4)
21-25	97 (55.7)	155 (49.8)
26-30	9(5.2)	17 (6.4)
>31	5 (2.9)	7 (2.3)
**Field of study**		
Medical and health sciences	102 (58.6)	134(43.0)
Engineering sciences	40 (22.9)	104 (33.5)
Social sciences	32(18.4)	73 (23.5)
**Type of higher institution**		
Private	150 (86.2)	273 (87.8)
Government	24(13.8)	38(12.2)
**Level of study**		
Diploma	40 (22.9)	54(17.4)
Undergraduate	122 (70.1)	224 (72.0)
Postgraduate	12 (6.9)	33 (10.6)
**Year of study**		
1^st^ year	51 (29.3)	143 (45.9)
2^nd^ year	39 (22.4)	73 (23.5)
3^rd^ year	49 (28.1)	64 (20.5)
4^th^ year	31 (17.81)	29 (9.3)
5 ^th^ year	4(2.3)	2 (0.6)

Of the 485 participants, 174 (35.9%) experienced migraine, while 311 (64.1%) did not. Of the 174 participants with migraine symptoms, 29 (16.7%) experienced pain since primary school, 73 (41.9%) since secondary school and 72 (41.4%) during pre-university/college/university. Further, 154 (88.5%) experienced migraine zero to three times per week; 15 (8.6%) had migraine four to six times a week; and 5 (2.9%) were unsure about the frequency per week. Conversely, 101 (58.0%) had migraine zero to three times per month; 32 (18.4%), four to six times per month; 13 (7.5%), seven to nine times per month; and 26 (15%), >10 times per month. Two (1.1%) were unsure of the frequency per month. Twenty (11.5%) participants were diagnosed with COVID-19 via PCR testing in October 2019, while 154 (88.5%) were not. Of these 154 participants, 43 (27.9%) were suspected to be in close contact with patients with COVID-19, and 111 (72.1%) were not diagnosed/never in contact with patients with COVID-19 (non-diagnosed COVID-19 periods).

**Figure 2:**
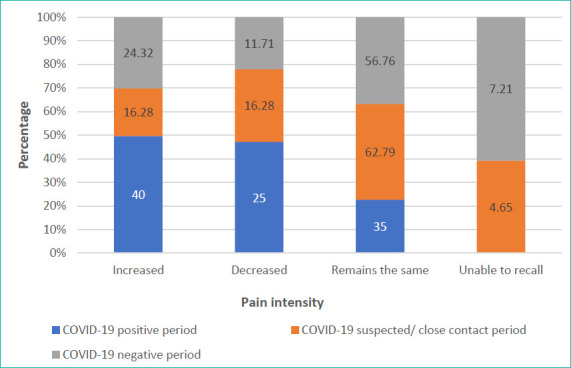
Migraine pain intensity during the COVID-19-positive, suspected/close contact and COVID-19-negative periods.

The clinical characteristics of migraine symptoms including changes in the frequency, intensity, severity and duration since October 2019 were analysed. Figure 2 depicts the migraine pain intensity during the COVID-19-positive, suspected/close contact and COVID-19-negative periods. Table 2 summarises the chi-square values and P-values of the clinical characteristics of migraine and COVID-19 diagnosis.

**Table 2:** Clinical characteristics of migraine based on the COVID-19 infection status of the participants (n=174).

Clinical characteristics	Diagnosed with COVID-19 (n=20)	Suspected to bein close contact with patients with COVID-19 (n=43)	Not diagnosed with COVID-19 (n=111)	χ^2^	P
	n (%)	n (%)	n (%)		
**Intensity**				9.013	0.173
Increased	8 (40)	7 (16.3)	27 (24.3)		
Decreased	5 (25)	7 (16.3)	13 (11.7)		
Remained the same	7 (35)	27 (62.7)	63 (56.8)		
Unable to recall	0	2(4.7)	8 (7.2)		
**Duration**				9.367	0.154
Increased	5 (25)	6 (14.0)	25 (22.5)		
Decreased	7 (35)	11 (25.6)	15 (13.5)		
Remained the same	7 (35)	23 (53.4)	57 (51.4)		
Unable to recall	1 (5)	3(6.9)	14 (12.6)		
**Frequency**				2.990	0.810
Increased	7 (35)	15 (34.9)	36 (32.4)		
Decreased	4 (20)	9 (21.0)	15 (13.5)		
Remained the same	6 (30)	14 (32.6)	48 (43.2)		
Unable to recall	3 (15)	5 (11.5)	12 (10.8)		
**Severity** Increased Decreased Remained the same Unable to recall	4 (20) 7 (35) 8 (40) 1 (5)	6 (14.0) 7 (16.3) 23 (53.4) 7 (16.3)	22 (19.8) 13 (11.7) 65 (58.6) 11 (9.9)	9.520	0.146

Among the 20 participants diagnosed with COVID-19, the most common trigger was a lack of sleep (75%), followed by stress (70%) and fatigue (50%). Among the 43 participants suspected to be in close contact with patients with COVID-19, the most common trigger was stress (83.7%), followed by a lack of sleep (76.7%) and fatigue (46.5%). Table 3 summarises the migraine triggers during the pandemic. For further analysis, the triggers were categorised under the following criteria: emotional stress (social isolation, stress or hormonal change), environmental factors (mask, infection or drugs), sleep pattern (lack of sleep or fatigue), weather changes (cold, sunlight or sensory stimuli) and combined triggers (combination of all including physical exercise, food intake and alcohol consumption).

**Table 3:** Migraine triggers during the COVID-19-positive and suspected/close contact periods among the participants (n=63).

Migraine triggers	COVID-19-positive period (n=20) n (%)	COVID-19 suspected/close contact period (n=43) n (%)
Social isolation	1(5)	12 (30.2)
Side effects of drugs consumed	1(5)	2(4.7)
Wearing of mask for a longer duration	4(20)	8(18.6)
Infection itself	7(35)	2(4.7)
Stress	14 (70)	36 (83.7)
Lack of sleep	15(75)	33 (76.7)
Fatigue	10(50)	20 (46.5)
Inadequate food intake	2(10)	4(9.3)
Physical exercise	3 (15)	4(9.3)
Hormonal change	3 (15)	11 (25.6)
Cold weather/environment	1(5)	9 (20.9)
Exposure to sunlight for a long period	3 (15)	12 (27.9)
Alcohol consumption	0(0)	2(4.7)
Various sensory stimuli (e.g. bright light or sound)	3 (15)	12 (27.9)
Others	1(5)	4(9.2)

**Figure 3:**
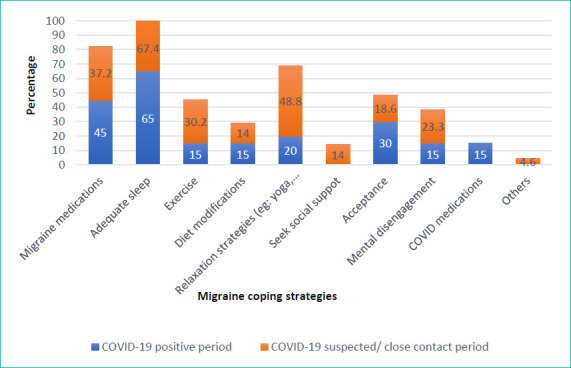
Coping strategies for migraine during the COVID-19-positive and suspected/close contact periods.

The coping strategies adopted by the participants diagnosed with COVID-19 were adequate sleep (65%), migraine medications (45%), acceptance (30%) and mental disengagement (15%). In comparison, the coping strategies applied by the participants suspected to be in close contact with patients with COVID-19 were adequate sleep (67.4%), relaxation exercise (48.8%), mental disengagement (23.3%) and acceptance (18.6%). Figure 3 summarises the coping strategies adopted by the university students to overcome their existing migraine symptoms during the pandemic. For further analysis, the coping strategies were categorised as follows: medication (migraine and COVID-19 medications), lifestyle changes (sleep, exercise, diet or relaxation), adaptive coping strategies (humanitarianism or assistance seeking), maladaptive coping strategies (acceptance or mental disengagement) and mixed strategies (combination of all). Table 4 summarises the categories of the triggers and coping strategies during the COVID-19-positive and suspected periods.

**Table 4:** Triggers of and coping strategies for migraine among the participants who were diagnosed with COVID-19 and suspected to be in close contact with patients with COVID-19 (n=63).

	Diagnosed with COVID-19 (n=20) n (%)	Suspected to be in close contact with patients with COVID-19 (n=43) n (%)	χ^2^	P
	n (%)	n (%)		
**Triggers**			5.019	0.285
Emotional stress	3 (15)	15 (34.9)		
Environmental factor	1 (5)	0 (0)		
Sleep pattern	6 (30)	8 (18.6)		
Weather change	1 (5)	3 (6.9)		
Combined trigger	9 (45)	17 (39.5)		
**Coping strategies**			7.641	0.106
Medications	2 (10)	2 (4.7)		
Lifestyle changes	7 (35)	23 (53.4)		
Maladaptive coping	5 (25)	2 (4.7)		
Mixed strategies	6 (30)	14 (32.5)		
Adaptive coping	0 (0)	2 (4.7)		

## Discussion

The study analysed the impact of the COVID-19 pandemic on existing migraine symptoms using a newly developed, validated and reliable instrument. The participants in the study exclusively included those exhibiting migraine symptoms. The assessment of pain intensity was based on the reported history of COVID-19 diagnosis since October 2019, which was regarded as either negative or positive during questionnaire completion. This allowed us to differentiate between the pain intensity associated with migraine and COVID-19 headaches. Of the 485 participants, 174 (35.9%) experienced migraines. These results are similar to the findings from other countries including Ethiopia (34%),^24^ and Southern Brazil (74.5%).^25^ A study conducted in more stressful periods would have revealed a higher prevalence of migraine headaches. The exact causal relationships in which stress causes migraine attacks and chronification or increases the burden of migraine remain unclear. Stress may induce sensitisation and altered cortical excitability, partially explaining attack triggering, the development of chronic migraine and increased burden of symptoms including interictal symptoms such as allodynia, photophobia or anxiety.^26^ Further, the methods used to determine the prevalence of migraine headaches can significantly affect the prevalence rates explaining the differences observed in epidemiological studies.^7^

Among the 20 participants diagnosed with COVID-19, the most common trigger was a lack of sleep (75%), followed by stress (70%) and fatigue (50%). Among the 43 participants suspected to be in close contact with patients with COVID-19, the most common trigger was stress (83.7%), followed by a lack of sleep (76.7%) and fatigue (46.5%). To our knowledge, our study is the first to identify triggers during the positive and suspected/close contact periods of infection. The results are similar to previous reports on migraine triggers during the COVID-19 pandemic.^9^ In both periods of infection, stress was found to be the most common trigger, followed by a lack of sleep. Further analysis revealed that the percentage of sleep (25%) and combined triggers (25%) in the group positive for COVID-19 and that of stress (23.2%) and combined triggers (20%) in the group suspected to be in close contact with patients with COVID-19 were higher among medical students. Stress is known as the most common trigger of migraine attacks. Furthermore, there is evidence that stress can help initiate migraine in individuals predisposed to the disorder and contribute to migraine chronification.^17^ As a global health crisis, the COVID-19 pandemic is perceived as a major stressful event, indicating that the pandemic has triggered migraine symptoms.

The coping strategies adopted by the participants diagnosed with COVID-19 were adequate sleep (65%), migraine medications (45%), acceptance (30%) and mental disengagement (15%). Conversely, the coping strategies adopted by those suspected to be in close contact with patients with COVID-19 were adequate sleep (67.4%), relaxation exercise (48.8%) and acceptance (18.6%). In the group diagnosed with COVID-19, the coping strategies applied were adequate sleep and acceptance. In the group suspected to be in close contact with patients with COVID-19, the most common coping strategy utilised was adequate sleep, followed by mental disengagement. Notably, social support seeking and humanitarianism are adaptive coping strategies, whereas acceptance and mental disengagement are maladaptive coping strategies. Based on these results, it can be concluded that university students in Malaysia use a maladaptive strategy rather than an adaptive strategy, especially those diagnosed with COVID-19. These findings align with those of a study conducted among 983 university students in Malaysia. A survey conducted online in 2020 showed that students used maladaptive coping strategies more than adaptive coping strategies to deal with anxiety caused by the pandemic and the effect of movement restriction. Social support seeking and acceptance were significantly associated with the level of anxiety. 14 This could be attributed to the cultural values of Asians. Rather than confronting their stressor(s), university students used a maladaptive coping strategy^27^ by accepting their current situation.

The clinical characteristics of migraine symptoms including the frequency, intensity, severity and duration since October 2019 were analysed. In all three groups, migraine frequency increased, similar to other reports.^28^ This frequency increase was accompanied by the overuse of medications in all groups, as explained by the coping strategies adopted. One possible reason could be general worries such as contagiousness, lockdown, social isolation and information overload.^28^ Another reason could be the relatively smaller sample size. In terms of migraine intensity, it increased only in the group positive for COVID-19, while it remained the same in the group suspected to be in close contact with patients with COVID-19 and group negative for COVID-19. The possible cause of increased intensity could be the presence of microorganisms that may activate inflammatory and nociceptive mediators that stimulate headache, such as nitric oxide, prostaglandins and cytokines.^29^ Conversely, the severity and duration of migraine remained the same in all groups. These results align with a previous report that close to a third (30%, n=1453) of individuals reported no perceived changes in pain severity.^30^ One of the possible reasons for such findings could be that the present study was conducted when students were still under a movement control order and preparing for the endemic period; hence, the data captured the overall recovery impact of the COVID-19 pandemic on these pain-related symptoms.

There are potential reasons why the triggers coping strategies and clinical characteristics of migraine did not show a significant association with COVID-19 diagnosis. The first reason is that the sample size of each group (n=20 for the group positive for COVID-19, n=43 for the group suspected to be in close contact with patients with COVID-19 and n=111 for the group negative for COVID-19) may not be large enough to detect a significant difference. The second reason could be the high variability within each group. The third reason could be the homogeneity of the symptoms.

While the study provides insights into the prevalence and associated factors of migraine symptoms among university students during the COVID-19 pandemic, there are a few limitations and recommendations. First, the study utilised convenience sampling, resulting in a limited generalisability of the findings to the broader population of university students. Second, the selfreported data may be subject to recall biases. Third, the cross-sectional design precludes establishing causal relationships, highlighting the need for longitudinal studies. Finally, other confounding factors such as comorbid medical conditions, medication use, lifestyle factors and environmental factors were not considered. These limitations indicate the necessity for healthcare providers to establish systematic screening protocols for migraine symptoms among university students to facilitate prompt identification and intervention. It is imperative to distinguish between migraine and COVID-19 headaches due to the symptom overlap to ensure precise diagnosis and treatment. Students ought to receive instruction on efficient coping mechanisms to handle migraine triggers, particularly within the pandemic context. This could entail stress management workshops, counselling services and healthy lifestyle changes.

## Conclusion

In conclusion, this cross-sectional study conducted among university students in Peninsular Malaysia revealed a considerable prevalence of migraine symptoms. During the pandemic, the common triggers for existing symptoms were stress and a lack of sleep (combined triggers). The coping strategy adopted by most university students was lifestyle changes. Conversely, the university students positive for COVID-19 applied maladaptive coping strategies, indicating the need for further attention. None of the triggers, coping strategies and clinical characteristics of migraine were significantly associated with COVID-19 diagnosis.

## Data sharing statement

The data are kept by the principal investigator and will be made available only upon request with permission from the investigators.

**How does this paper make a difference in general practice?**

- The study provides information on the existing pattern of migraine symptoms and triggers, enabling healthcare providers to better diagnose and treat patients and thereby improve their quality of life.
- Understanding the triggers and risk factors of migraine can help general practitioners to educate patients about lifestyle modifications.
- The findings can inform guidelines on the management of migraine.
- Migraine can substantially affect mental health. This research helps in recognising and addressing the mental health challenges faced by university students experiencing migraine.
- The research conducted during the COVID-19 pandemic highlights the importance of healthcare system preparedness for pandemics and public health emergencies.
